# Coupled Impact of Anthocyanin and Mineral Concentrations in Cranberry Juice on Gut Microbiota and Function Modulation: A First Demonstration

**DOI:** 10.3390/molecules30193986

**Published:** 2025-10-04

**Authors:** Eva Revellat, Joanie Dupont-Morissette, Thibault V. Varin, Geneviève Pilon, André Marette, Laurent Bazinet

**Affiliations:** 1 Institute of Nutrition and Functional Foods (INAF) and Department of Food Sciences, Université Laval, Québec, QC G1V OA6, Canada; eva.revellat.1@ulaval.ca (E.R.); andre.marette@criucpq.ulaval.ca (A.M.); 2 Laboratoire de Transformation Alimentaire et Procédés ElectroMembranaires (LTAPEM, Laboratory of Food Processing and ElectroMembrane Processes), Université Laval, Québec, QC G1V OA6, Canada; 3 Heart and Lung Institute, Department of Medicine, Université Laval, Québec, QC G1V 4G5, Canada; joanie.dupont-morissette@criucpq.ulaval.ca (J.D.-M.); thibaut.varin.1@ulaval.ca (T.V.V.); genevieve.pilon@criucpq.ulaval.ca (G.P.)

**Keywords:** cranberry juice, anthocyanin, minerals, microbiota, modulation

## Abstract

Cranberry juice (CJ), a natural source of anthocyanins, may provide additional health benefits when enriched, as anthocyanins have been shown to influence gut microbiota composition. This study investigated the effects of varying anthocyanin and mineral concentrations in CJ on gut microbiota in mice. Using electrodialysis with filtration membranes (EDFM), five CJ samples with different anthocyanin/mineral enrichment levels (0/0, −31/−85%, −19/−70%, 26/−32%, and 44/−60%) were produced and administered to C57BL/6J mice for four weeks. Gut microbiota composition was analyzed via 16S rRNA sequencing, and inflammation was determined in macroscopic observations of intestinal tissues. While α and β diversity remained unchanged, differential abundance analysis revealed that gut microbiota changes were influenced by anthocyanin and mineral concentrations. Synergistic trends were observed for *Colidextribacter* and *Oscillibacter* (increasing with both compounds) and for *Turicibacter*, *Romboutsia*, *Enterorhabdus*, and *Bifidobacterium* (decreasing with both compounds). Antagonistic trends emerged for *Dubosiella*, *Acetatifactor*, *A2*, *Ruminococcus*, and *Intestinimonas* (decreasing with anthocyanins and increasing with minerals), and the reverse was found for *Ligilactobacillus*. The most significant microbiota shifts occurred with the −31/−85% CJ, suggesting a strong effect of its low anthocyanin and mineral content. But further analysis is needed to assess their metabolic effects and impact on intestinal health.

## 1. Introduction

Cranberry juice is a rich source of bioactive compounds, particularly anthocyanins, which have demonstrated potential for modulating gut microbiota composition. Early investigations into cranberry extract (CE) supplementation revealed a significant impact on the gut microbiota of high-fat high-sugar (HFHS)-fed mice, notably increasing the abundance of *Akkermansia* spp. [1]. This effect was linked to enhanced mucin production by proanthocyanidins, providing a favorable environment for *Akkermansia* to thrive [2]. A review has detailed subsequent studies reporting similar findings [3], reinforcing the role of cranberry polyphenols in gut microbiota modulation. Polyphenols have recently been described as “duplibiotics,” reflecting their dual role as unabsorbed substrates capable of modulating gut microbiota through both antimicrobial and prebiotic mechanisms [4]. Beyond cranberry extracts, research has specifically examined the effects of cranberry juice on gut microbiota composition. In vitro studies [5,6] and clinical trials [7,8,9,10,11,12] have consistently reported microbiota-modulating effects, highlighting an increase in beneficial bacteria such as *Bifidobacterium*, alongside a reduction in potentially harmful bacterial taxa. Given the high anthocyanin content of cranberry juice, recent investigations have explored the impact of anthocyanin supplementation from various dietary sources on gut microbiota modulation in rodent models.

Anthocyanins consist of an aglycone (anthocyanidin) conjugated to a sugar moiety, and cranberry juice contains six major anthocyanins based on peonidin and cyanidin linked to galactose, arabinose, or glucose [13,14]. While a small fraction of anthocyanins is absorbed in the stomach, their low systemic bioavailability suggests that colonic metabolism plays a key role in their physiological effects [15]. Unabsorbed anthocyanins undergo bacterial biotransformation, potentially enhancing their bioavailability and bioactivity in target organs and cells [15]. For instance, in vivo studies have demonstrated that cranberry anthocyanin extract supplementation leads to a reduction in *Rikenella* and *Rikenellaceae* abundance [16].

Recent in vitro, in vivo, and clinical studies have further underscored the role of anthocyanins in shaping gut microbiota composition. While not exclusive to cranberries, research on anthocyanin-rich berries such as black cherries and blueberries has shown that anthocyanin supplementation promotes the growth of beneficial bacteria, including *Akkermansia*, *Bifidobacterium*, *Lactobacillus*, and certain *Actinobacteria* families [15]. A recent meta-analysis has also suggested that anthocyanin supplementation ameliorates gut health biomarkers in rodent models by alleviating obesity-induced gut dysbiosis [17]. Collectively, these findings highlight the specific contribution of anthocyanins, beyond polyphenols as a whole, in shaping gut microbial composition, and reinforce the importance of investigating cranberry anthocyanins in this context.

However, cranberry juice consumption has been associated with gastrointestinal discomfort, likely due to its organic acids, which have been shown in vitro to induce inflammation [18]. But, a study in mice with deacidified cranberry juice did not report inflammation [5], suggesting that polyphenols may counteract the negative effects of organic acids. Furthermore, cranberry juice also contains significant amounts of minerals, including potassium (62%), followed by chlorine, calcium, phosphorus, magnesium, and sodium [19]. Recent studies have highlighted the antagonistic effect of potassium supplementation on the development of inflammatory diseases related to high salt intake [20]. And lately, studies have reported the modulation of gut microbiota by potassium in a traditional Chinese diet among healthy individuals, as well as the effects of potassium-rich coconut water on patients with ulcerative colitis, leading to an increased abundance of health-associated genera [21,22]. Given that most cranberry fruit is processed into juice [23], cranberry juice could be easily incorporated into diets, offering a practical alternative to supplementation and facilitating clinical trials. Recently, electrodialysis with filtration membrane (EDFM) has enabled the enrichment and demineralization of cranberry juice [24]. This technique allows the production of juices with varying concentrations of anthocyanins and minerals, without altering the organic acid content. This offers the opportunity to conduct targeted studies on the individual and combined impact of anthocyanins and minerals on gut microbiota modulation. In this context, the principal objective of the present study was to assess the impact of daily CJ supplementation with various anthocyanin and mineral contents on gut health as determined by gut microbiota composition and intestinal inflammation.

## 2. Results

### 2.1. Effect of CJ on the Food Intake and Body Weight

**Figure 1:**
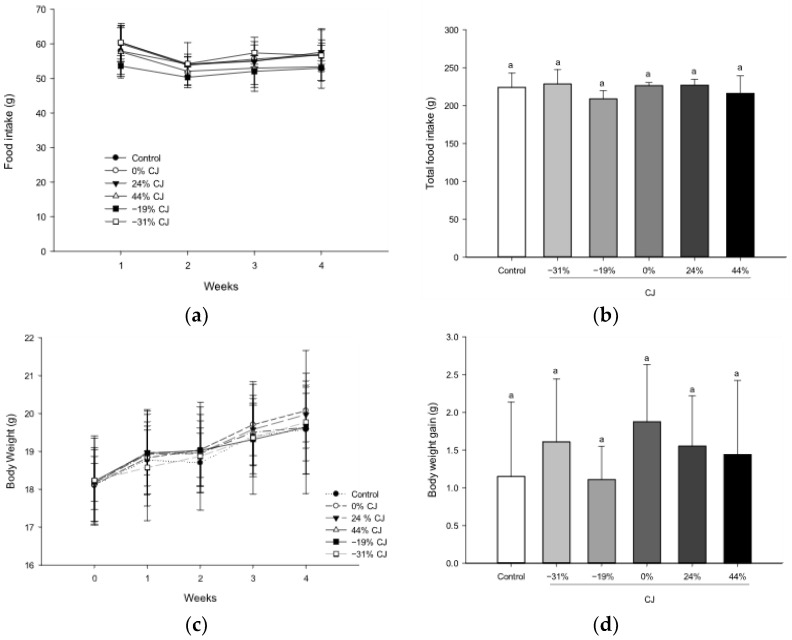
Effect of cranberry juice (CJ) anthocyanin content on the (**a**) food intake, (**b**) total food intake, (**c**) body weight, and (**d**) total body weight gain in mice in comparison with water (control). For (**a**,**b**), results are presented as the mean ± SEM of 4 batches. For (**c**,**d**), results are presented as the mean ± SEM and *n = 12*. Statistical analysis was performed using one-way ANOVA followed by Tukey’s post hoc test (*p* < 0.05). For (**a**,**c**), the absence of a letter means that there is no statistically significant effect at a probability level of 0.05. For (**b**,**d**), the same letter means that the results are not significantly different at a probability level of 0.05. For (**c**), the body weight of T0 was measured on Tuesday and then each Thursday for T1, T2, T3, and T4.

The food intake of each cage was measured at T1, T2, T3, and T4, and no significant impact of the concentration of anthocyanins on the CJ was highlighted (Figure 1a). Furthermore, the total food intake was similar between groups (Figure 1b). Body weight was also measured during the 4 weeks, and no significant difference in body weight was observed at each time between groups (Figure 1c). Also, the total body weight gain was similar between groups (Figure 1d).

### 2.2. Feces and Post-Mortem Observations

*Hemoccult*

All throughout the 4 weeks of treatment, there was no presence of occult blood (OB) in the feces of the −31, −19, 0, 26, and 44% CJ groups and in the feces of the control group.

*Organ weights*

**Figure 2:**
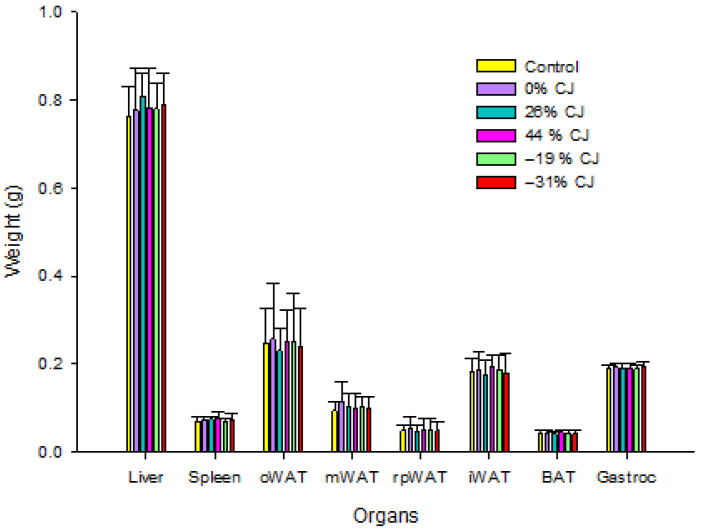
Effect of cranberry juice (CJ) anthocyanin content on the weight (g) of the Owat, mWAT, rpWAT, iWAT, Bat, and Gastroc. Results are presented as means ± SD for each group (*n = 12*). Statistical analysis was performed using one-way ANOVA followed by Tukey’s post hoc test (*p* < 0.05). For each organ, there was no statistically significant difference between the groups at a probability level of *p* < 0.05.

The weights of the liver, spleen, Owat (ovarian white adipose tissue), mWAT (mesenteric white adipose tissue), rpWAT (retroperitoneal white adipose tissue), Iwat (inguinal white adipose tissue), Bat (brown adipose tissue), and Gastroc were not different between treatments (Figure 2).

*Intestinal macroscopic observation*

The duodenum, jejunum, ileum, and colon of each mouse of each group were macroscopically observed to determine any inflammation, thickness, and vascularization changes between groups (Table 1) as previously described [5]. There was no significant difference between the groups.

### 2.3. Impact of CJ Administration and Anthocyanin Concentration in CJ on the Gut Microbiota

**Figure 3:**
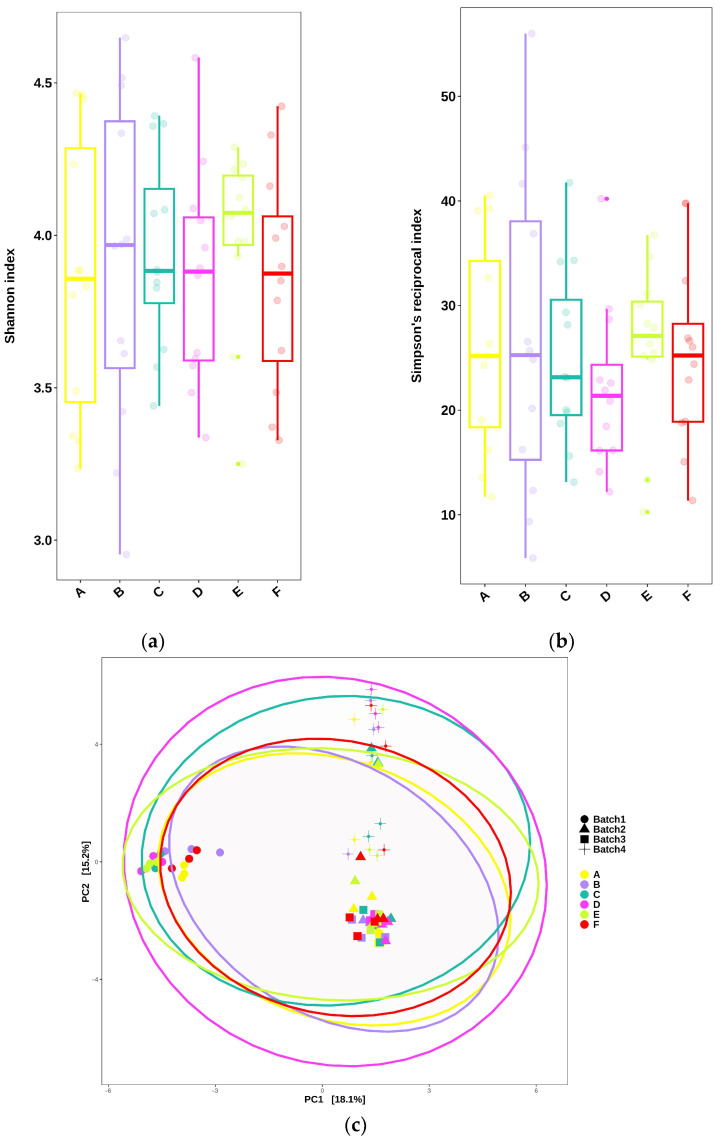
Impact of anthocyanin concentration in cranberry juice (CJ) on the alpha-diversity of the gut microbiota after the end of the experiments (T4) for each group, as measured by (**a**) the Shannon index or (**b**) Simpson‘s reciprocal index. Statistical comparisons between groups were performed using one-way ANOVA; no significant differences were detected (*p* > 0.05). (**c**) The beta-diversity of the gut microbiota after the end of the experiment (T4) between groups, as visualized by the principal component analysis (PCA). Seventy-two female C57BL/6J mice were divided into six groups (Control, A–F; twelve mice/group). Each group comprised four batches (Batches 1–4) of three mice per cage. Group identification: (A) water (control group), (B) 0% CJ, (C) 26% CJ, (D) 44% CJ, (E) −19% CJ, and (F) −44% CJ.

The impact of the different concentrations of anthocyanins in CJ was assessed by collecting the fecal samples at T4. The Shannon and Simpson reciprocal indexes for each group were determined to estimate the alpha-diversity (Figure 3a,b). Simpson’s reciprocal index gives more weight to the more abundant species in a sample. The diversity indexes did not increase after 4 weeks of receiving the different CJ compared to the groups receiving water (control group). Furthermore, there was no distinct separation between the microbial communities of the different groups, as testified by the principal component analysis (PCA) (Figure 3c). However, an important batch effect was observed despite similar lab and manipulation conditions for each batch. The differential abundance analysis was adjusted accordingly to account for it [25].

#### 2.3.1. Effect of CJ Administration on the Composition of the Gut Microbiota

**Figure 4:**
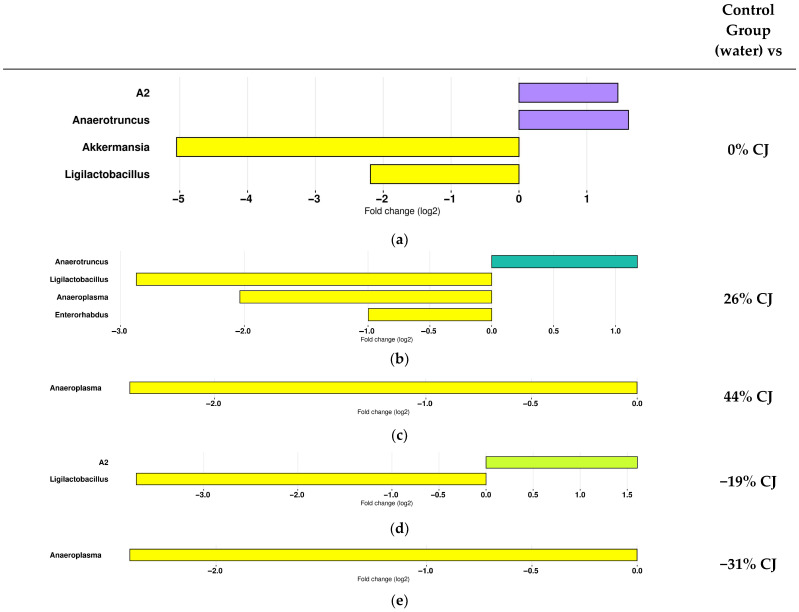
Administration of cranberry juice (CJ) with different anthocyanin concentrations is associated with changes in the gut microbiota of mice in comparison with the control (water). Log2 fold changes of bacterial genera with significant differential abundance between mice treated with (**a**) water (yellow) and 0% CJ (purple), (**b**) water and 26% CJ (blue), (**c**) water and 44% CJ (**d**) water and −19% CJ (green), and (**e**) water and −31% CJ.

Differential abundance analysis on T4 highlighted significant differences in the gut microbial communities of the CJ-treated mice from those of control mice (water) (Figure 4). The results of differential abundance analysis demonstrated that the CJs induced changes in the gut microbiota composition at the genus level. When CJ was administered, *A2* and *Anaerotruncus* abundance increased, whereas *Ligilactobacillus*, *Anaeoroplama*, *Akkermensia*, and *Enterorhabdus* were decreased (Figure 4).

Higher abundance of the genera *A2* and *Anaerotruncus* and a lower representation of *Akkermensia* and *Ligilactobacillus* were observed in the 0% CJ-treated mice compared to the control mice (water) (Figure 4a). Similarly, the gut microbial community of the 26% CJ-treated mice was characterized by an increased abundance of *Anaerotruncus* but by a lower representation of *Ligilactobacillus*, *Anaeroplama*, and *Enterorhabdus* (Figure 4b). Furthermore, an increased abundance of *A2* and a reduction in *Ligilactobacillus* were identified in the −19% CJ-treated mice compared to the control mice (water) (Figure 4d). Finally, overrepresentation of the genus *Anaeroplasma* was also identified as the main feature discriminating control (water) mice from the 44% CJ-treated mice microbiota (Figure 4c) and from the −31% CJ-treated mice (Figure 4e).

#### 2.3.2. Effect of Anthocyanin Concentration on the Composition of the Gut Microbiota

The effect of the level of anthocyanin enrichment or impoverishment of CJ on the composition of the gut microbiota was also investigated, comparing the gut microbiota composition of the most anthocyanin impoverished (−31%) CJ-treated mice to the −19, 0, 26, and 44% CJ groups. This approach was selected, as the extreme impoverishment levels provide the most significant insights into the impact on gut microbiota composition.

The results of differential abundance analysis demonstrated that the −31% CJs induced the most changes in the gut microbiota composition in comparison with other groups. When −31% CJ was administered *Akkermansia*, *Bifidobacterium*, *Clostridium sensu stricto 1*, *Enterorhabdus*, *Family XIII AD3011 group*, *Dorea*, *Dubosiella*, *Lactobacillus, Romboutsia*, and *Turicibacter* abundance increased, whereas *Anaeroplasma*, *Oscillibacter*, *Acetatifactor*, *Intestinimonas*, and *Colidextribacter* were less abundant.

**Figure 5:**
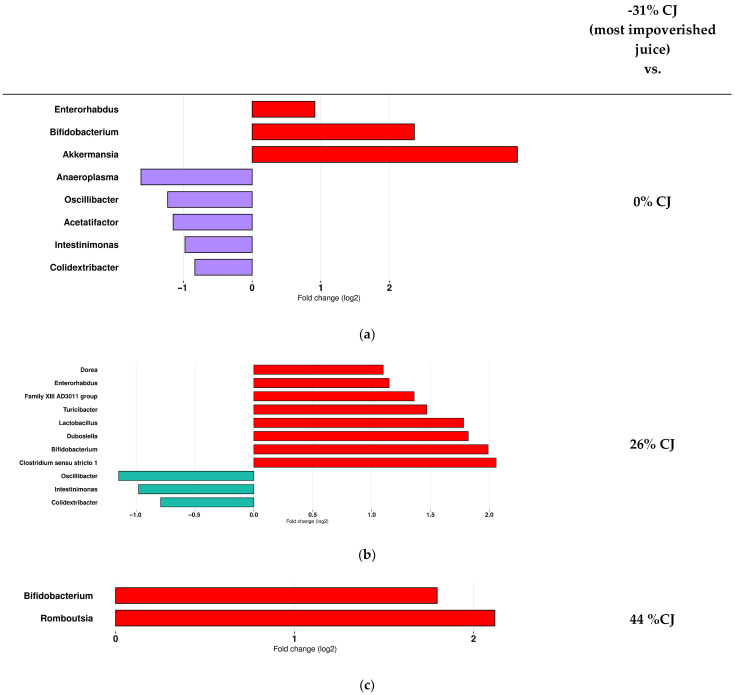
Administration of cranberry juices (CJs) with different anthocyanin concentrations is associated with changes in the gut microbiota of mice in comparison with the most anthocyanin-impoverished CJ. DESeq2 was used in order to explore the taxa (at the genus level) that more strongly discriminated between mice treated with (**a**) −31% CJ (red) and 0% CJ (purple), (**b**) −31% CJ (red) and 26% CJ (blue), and (**c**) −31% CJ (red) and 44% CJ No significant results discriminating the gut microbiota of mice treated with the −31% CJ and −19% CJ were identified.

Hence, there was an increase in *Enterorhabdus, Bifidobacterium*, and *Akkermensia* and a lower abundance of *Anaeroplasma*, *Oscillibacter*, *Acetatifactor*, *Intestinimonas*, and *Colidextribacter* in the −31% CJ-treated mice compared to the 0% CJ-treated mice (Figure 5a). Furthermore, there was an increase in *Dorea, Enterorhabdus*, *Family XIII AD3011 group*, *Turicibacter*, *Lactobacillus*, *Dubosiella*, *Bifidobacterium*, and *Clostridium sensu stricto 1*, and a lower representation *of Oscillobacter*, *Intestinimonas*, and *Colidextribacter* for the −31% CJ-treated mice compared to the 26% CJ-treated mice (Figure 5b). Finally, at T4, overrepresentation of the genera *Bifidobacterium and Romboutsia* was identified as the main feature discriminating the −31%-CJ-treated mice microbiota from the 44% CJ-treated mice (Figure 5c).

**Figure 6:**
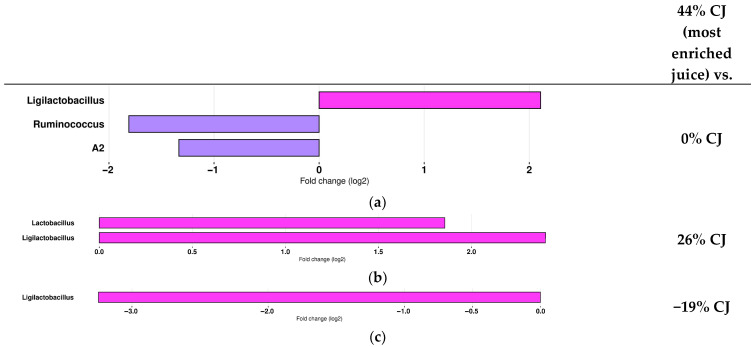
Administration of cranberry juices (CJs) with different anthocyanin concentrations is associated with changes in the gut microbiota of mice in comparison with the most anthocyanin-enriched CJ. Log2 fold changes of bacterial genera with significant differential abundance ibetween mice treated with (**a**) 44% CJ (pink) and 0% CJ (purple), (**b**) 44% CJ (pink) and 26% CJ, and (**c**) 44% CJ (pink) and −19% CJ.

The effect of the level of anthocyanin enrichment or impoverishment of CJ on the composition of the gut microbiota was also investigated, comparing the gut microbiota composition of the most anthocyanin-enriched (44%) CJ-treated mice to the −19, 0, and 26% CJ groups (Figure 6). This approach was selected once more, as the extreme enrichment level provides the most significant insights into the impact on gut microbiota composition.

The results of differential abundance analysis demonstrated that the 44% CJ induced changes in the gut microbiota composition. When 44% CJ was administered, *Lactobacillus* and *Ligilactobacillus* abundance increased, whereas *A2* and *Ruminococcus* decreased. More specifically, at T4, there was an increased representation of the genera *Ruminococcus* and *A2* in the 0% CJ-treated mice vs. the 44% CJ-treated mice (Figure 6a). The genus *Lactobacillus* was more abundant in the 44% CJ-treated mice vs. the 26% CJ-treated mice (Figure 6b). The presence of *Ligilactobacillus* was increased in the 44% CJ-treated mice compared to the 0% CJ-treated mice and the 26% CJ-treated mice (Figure 6a,b), but an overrepresentation of the genus *Ligilactobacillus* was also identified as the main feature discriminating 44% CJ-treated mice from the −19% CJ-treated mice (Figure 6c).

### 2.4. Impact of CJ Administration and Anthocyanin Concentration in the CJ on the Functional Prediction Pathways of the Gut Microbiota

**Figure 7:**
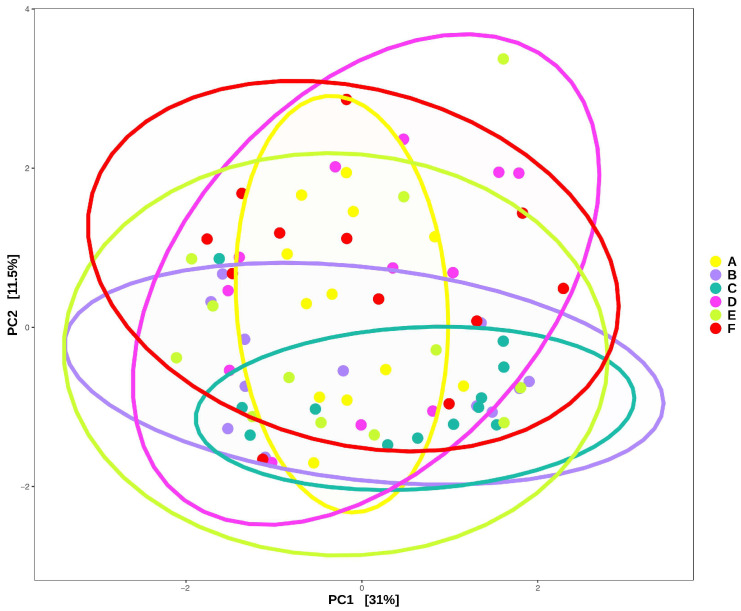
Impact of the anthocyanin concentration in cranberry juice (CJ) on the functional prediction after 4 weeks of treatment of the gut microbiota, reflected by the PCA.

Principal component analysis (PCA) revealed no distinct separation between the functional prediction of the different groups (Figure 7). The batch effect was less pronounced (as metabolic functions are more preserved), and groups were more separated in the functional analysis than in the PCA based on taxa. The juice diet appeared to influence microbial functions.

#### 2.4.1. Effect of the CJ Administration on the Functional Pathways of the Gut Microbiota

**Figure 8:**
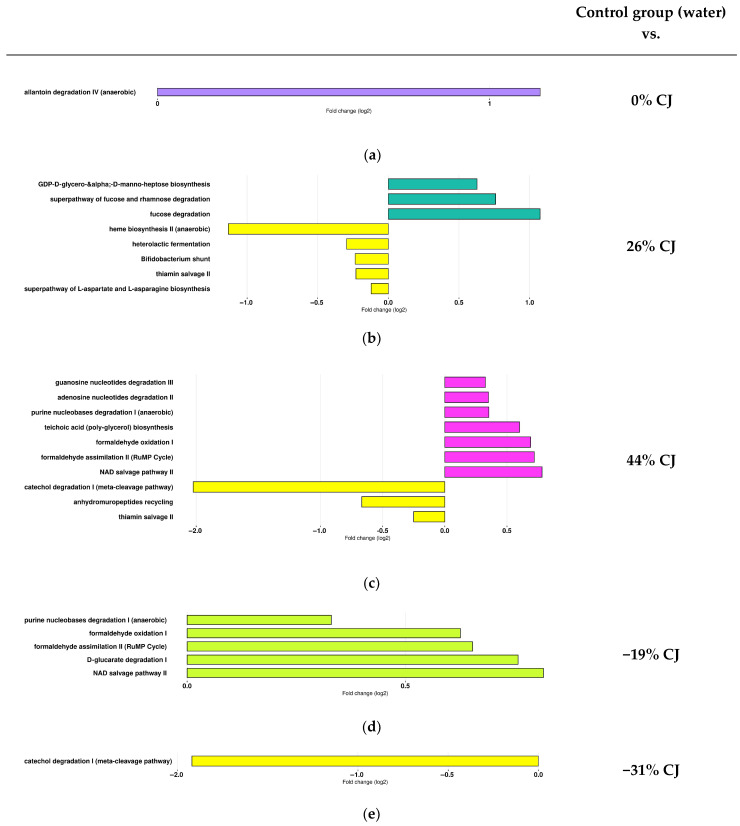
Administration of cranberry juices (CJs) with different anthocyanin concentrations is associated with changes in the gut microbial functional pathways of mice in comparison with control mice (water). DESeq2 was used in order to explore the microbial pathways that more strongly discriminated between mice treated with (**a**) water and 0% CJ (purple), (**b**) water (yellow) and 26% CJ (blue), (**c**) water and 44% CJ (pink), (**d**) water and −19% CJ (green), and (**e**) water and −31% CJ.

PICRUSt2 was used to assess whether the different cranberry juices (CJs) caused functional changes in the gut microbiome compared to the control (water). The results showed that CJs led to functional alterations in the gut microbiome. Specifically, 19 pathways were affected: 12 pathways were more prevalent in the groups receiving CJs compared to the control group, while 7 pathways were more abundant in the control group than in the CJ-treated groups (Figure 8). These pathways covered a diverse range of categories, including degradation and catabolism, biosynthesis, fermentation and energy production, formaldehyde metabolism, cofactor and vitamin metabolism, and cell wall recycling. Hence, functions associated with allantoin degradation IV were more represented in the gut microbiota of the 0% CJ-treated mice than in control mice (Figure 8a). Nucleotide-activated sugar biosynthesis (e.g., GDP-D-glycero-&alpha and -D-manno-heptose biosynthesis) and carbohydrate degradation (e.g., fucose and rhamnose degradation) pathways were more represented in the gut microbiota of the 26% CJ-treated mice than the control mice (Figure 8b). In comparison, in the gut microbiota of the control group (water), amino acid and heme biosynthesis (e.g., superpathway of L-arpartate and L-asparagine biosynthesis and heme biosynthesis II), carbohydrate metabolization (e.g., heterolactic fermentation and Bifidobacterium shunt), and vitamin salvage (e.g., thiamin salvage II) pathways were more represented. Nucleotide degradation, cofactor metabolization (e.g., adenosine and guanine degradation and NAD salvage pathway), polysaccharide biosynthesis (e.g., teichoic acid and poly-glycerol), and formaldehyde metabolization (oxidation and assimilation) pathways were more abundant in the 44% CJ-treated mice compared to the control mice (Figure 8c). In contrast, pathways such as vitamin salvage (thiamin salvage II), catechol degradation, and recovery and reuse of peptidoglycan products (e.g., anhydromuropeptides recycling) were more represented in the gut microbiota of the control group (water). The gut microbiota of the −19% CJ-treated mice revealed a higher abundance of nucleotide and organic acid degradation (e.g., purine nucleobase degradation and D-glutarate degradation I), formaldehyde metabolization (oxidation and assimilation), and NAD salvage pathways compared to the control mice (Figure 8d). Functions associated with catechol degradation were more represented in the gut microbiota of the control group than in the gut of the −31% CJ-treated mice (Figure 8e).

#### 2.4.2. Effect of Anthocyanin Concentration in the Juice on the Functional Predictions of the Gut Microbiota

The impact of the concentration of anthocyanins in the CJ on the predicted functions of the gut microbiota was compared first to the most impoverished juice (−31%) CJ-treated mice and the −19, 0, 26, and the 44% CJ groups. The results showed that −31% CJs led to the most important functional alterations in the gut microbiome. Specifically, 83 pathways were identified as significant: 53 pathways were more abundant in the group receiving −31% CJ compared to the other CJ, while 30 pathways were more represented in other groups than in the −31% CJ-treated group. These pathways covered a diverse range of categories, including fermentation, nucleotide and amino acid biosynthesis, vitamin and cofactor biosynthesis, degradation, and glycolysis.

**Figure 9:**
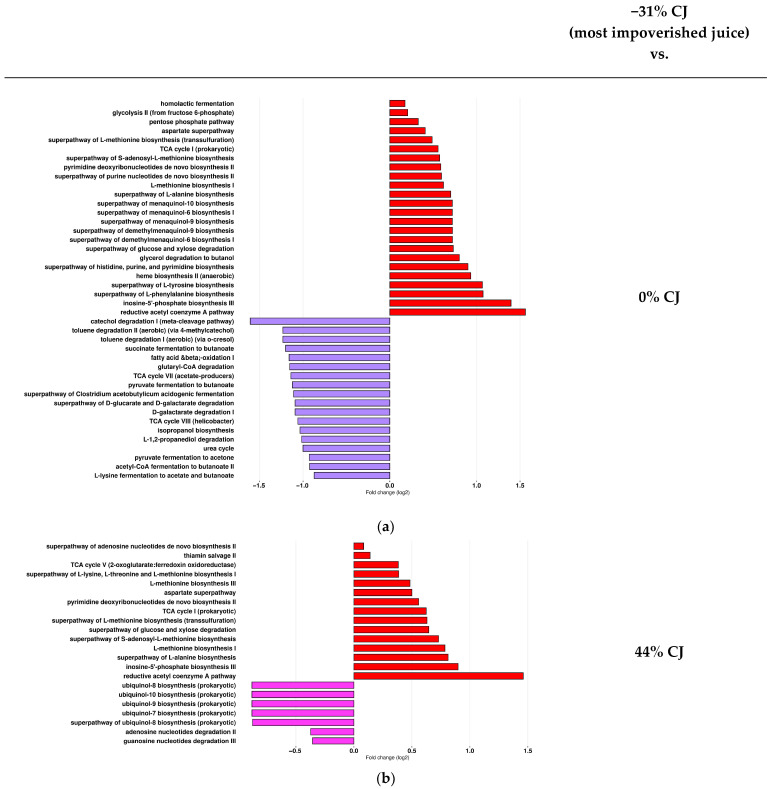
Administration of cranberry juices (CJs) with different anthocyanin concentrations is associated with changes in the gut microbial functional pathways of mice in comparison with the most anthocyanin-impoverished CJ. DESeq2 was used in order to identify the microbial pathways that more strongly discriminated between mice treated with (**a**) −31% CJ (red) and 0% CJ (purple) and (**b**) −31% CJ (red) and 44% CJ (pink).

The gut microbiota of the −31% CJ-treated mice compared to the 0% CJ-treated mice was enriched in menaquinol, amino acid biosynthesis (e.g., superpathway of menaquinol-10 biosynthesis, as well as methionine, tyrosine, and phenylalanine biosynthesis), sugar degradation (glucose and xylose degradation), and generation of precursor metabolites and energy (pentose phosphate) (Figure 9a). In contrast, in the gut microbiota of the 0% CJ-treated mice compared to the −31% CJ-treated mice, aromatic compound, amino acid degradation (e.g., catechol degradation, toluene degradation, and Acetyl-CoA degradation), organic acid, amino acid fermentation (succinate and lysine fermentation to butanoate), and short-chain fatty acid (SCFA) biosynthesis (acetate and butanotate) pathways were more represented (Figure 9a). Similar results were obtained comparing pathways representative of the −31% CJ-treated mice and the 26% and −19% CJ-treated mice and are detailed in the Supplementary Materials (Figure S1a,b). Similarly, in the gut microbiota of the −31% CJ-treated mice, amino acid biosynthesis (e.g., alanine biosynthesis) pathways were more abundant compared to the 44% CJ-treated mice (Figure 9b). But, in the gut microbiota of the 44% CJ-treated mice compared to the −31% CJ-treated mice, nucleotide degradation (e.g., guanosine degradation) and ubiquinol biosynthesis pathways, which play a critical role in cellular respiration and energy production, were more represented (Figure 9b).

The impact of the concentration of anthocyanin in the CJ on the functional prediction of the gut microbiota was compared between each group. The differential abundance analysis only showed microbial pathways that strongly discriminated between the gut microbiota of mice treated with 26% CJ and the −19% and 44% CJ-treated mice, and this is detailed in the Supplementary Materials (Figure S2).

## 3. Discussion

### 3.1. Impact of Anthocyanin Concentration on the Physiological Parameters and Inflammatory State

The effects of daily CJ supplementation and anthocyanin concentration on physiological parameters and the onset of intestinal inflammation were explored. No changes were observed in food intake or body weight among the mice, regardless of the anthocyanin enrichment. Polyphenols (including anthocyanins) have been reported to enhance satiety, potentially influencing these parameters [26,27]. However, similar findings were previously reported for cranberry juice consumption [5]. Additionally, 0% CJ did not lead to the presence of OB in the feces, nor did −31% and −19% CJ, which is consistent with the findings in a previous study on the effects of deacidified juice on inflammatory states [5]. While in vitro studies have suggested that juice could induce gut inflammation [28], the polyphenol content seems to provide intestinal protection. This protective effect has also been observed in diet-induced obesity models, where inflammation was primarily driven by the diet [29,30]. Furthermore, post-mortem analyses revealed no significant changes in organ weights for all groups, and macroscopic examinations of the duodenum, jejunum, ileum, and colon in all groups showed no signs of inflammation, thickening, or increased vascularization, with no differences between the control and all groups, aligning with previous findings [5]. The reduction in anthocyanin content did not lead to intestinal inflammation. These results confirmed that other polyphenols may play a protective role. CJ contains proanthocyanidins (PACs), which are well-documented in the literature for their protective effects against intestinal inflammation [31,32]. Additionally, CJ is known to contain various other polyphenols that could significantly contribute to preventing intestinal inflammation [33,34,35]. A previous study on deacidified CJ further supports this hypothesis. The authors compared two different CJ lots from distinct harvests and climate conditions. Daily consumption of 7°Brix CJ induced intestinal inflammatory symptoms in mice, while 0% deacidified CJ (DCJ), from a different lot, did not. Notably, the 0% DCJ had higher levels of anthocyanins, PACs, and other polyphenols compared to the 7°Brix CJ [5]. This suggests that, within the range of anthocyanin enrichment and depletion studied presently, anthocyanins alone were not the primary molecules responsible for protecting against intestinal inflammation.

### 3.2. Impact of Anthocyanin Concentration in the Cranberry Juice on the Gut Microbiota

The presence of *Ligilactobacillus* increased in the mice treated with 44% anthocyanin-enriched CJ compared to the 0% CJ-treated mice, suggesting a potential positive effect of anthocyanins on its abundance, as already demonstrated in the literature for most of the *Lactobacillus* genera [15]. However, compared to the control group, the presence of *Ligilactobacillus* decreased when CJs (0%, −19%, and 26%) were administered. This may indicate that another component of the 44% CJ offsets the negative impact observed with the 0% CJ. Given that EDFM treatment led to demineralization, these results suggest that minerals may also influence *Ligilactobacillus* abundance. Similarly, the presence of *Akkermansia* decreased when the 0% CJ was administered, compared to the water-treated group. Interestingly, *Akkermansia* abundance increased in the group treated with −31% CJ compared to the 0% CJ-treated group, suggesting a possible negative impact of this juice despite the known beneficial effect of anthocyanins on *Akkermansia* [15], suggesting that minerals in the juice lead to a decrease in *Akkermensia* in the 0% CJ-treated group. The −31% CJ might have counteracted the negative effects observed with 0% CJ, as it contains lower anthocyanin and mineral levels. In addition, the abundance of *A2* increased when 0% and −19% CJs were administered compared to the water-treated group, but increased in the 0% CJ group compared to the 44% CJ group. These findings indicate that anthocyanins alone may not drive the increase in *A2* abundance, and that minerals in the 0% CJ might have a positive impact. Furthermore, the presence of *Enterorhabdus* increased in the mice treated with −31% anthocyanin-enriched CJ compared to the 0 and 26% CJ-treated mice, suggesting a potential negative impact of anthocyanins on this genus abundance. And compared to the control group, *Enterorhabdus* abundance decreased when 26% CJ was administered. This may indicate that *Enterorhabdus* is negatively correlated to anthocyanin and mineral content in the juice. Also, *Anaeroplasma* increased in the gut of the mice treated with the 0% juice compared to the −31% CJ, indicating a positive impact of anthocyanins on *Anaeroplasma* abundance. However, compared to the control group, *Anaeroplama* decreased when treated with the 26% CJ. This may indicate that another component of the 0% CJ offsets the negative impact of the −31% CJ. As EDFM treatment led to demineralization, and the −31% CJ was the most demineralized juice, these results suggest that minerals could increase genus abundance.

Overall, there was no clear trend indicating that changes in anthocyanin concentration in the juice led to an increase in the abundance of specific bacterial genera. However, the EDFM process resulted in juice demineralization, suggesting that minerals, either independently or synergistically, played a role in gut microbiota modulation. This will be discussed in the following section.

### 3.3. Combined Effect of Minerals and Anthocyanins on the Gut Microbiota—Synergistic and Antagonistic Effects of Anthocyanins and Minerals

**Figure 10:**
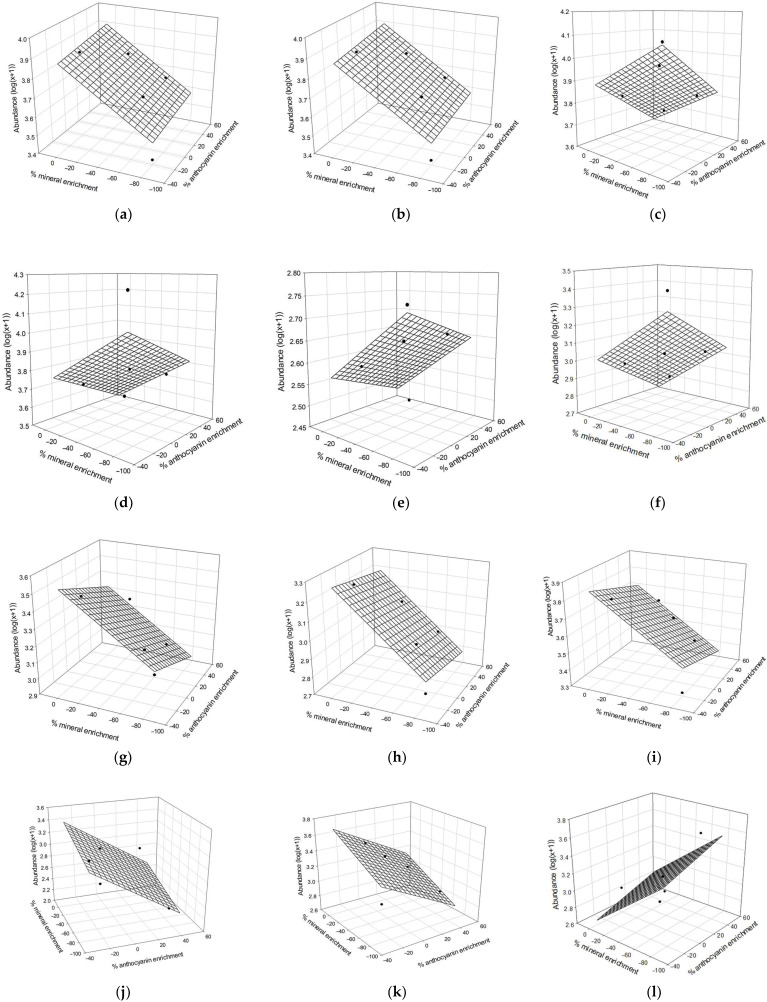
Three-dimensional representation of relative abundance of (**a**) *Colidextribacter*, (**b**) *Oscillibacter*, (**c**) *Turicibacter*, (**d**) *Bifidobacterium*, (**e**) *Enterorhabdus*, (**f**) *Romboutsia*, (**g**) *Ruminococcus*, (**h**) *Acetatifactor*, (**i**) *Intestinimonas*, (**j**) *Dubosiella*, (**k**) *A2*, and (**l**) *Ligilactobacillus* as a function of mineral and anthocyanin enrichments.

In order to estimate the impact of anthocyanin and mineral levels on the gut microbiota composition, a 3D representation and a plane equation (Z = zo + ax + by) were chosen to fit the data (Figure 10). Only bacterial genera with R^2^ values ≥ 0.47 were retained to highlight potential trends and to focus on the most relevant findings (Table 2). Although some of the individual coefficients were not statistically significant, these trends may provide preliminary insights into how bacteria might respond to such compositional changes. In this exploratory context, this analysis allowed for clustering bacteria depending on the positive or negative impact of mineral and anthocyanin contents on genus abundance. Using the different slopes (Table 2), four different bacterial groups were differentiated in accordance with the mineral and anthocyanin enrichment. Group 1 (*Colidextribacter* and *Oscillibacter*) showed positive synergistic effects of both minerals and anthocyanins on bacterial abundance. In Group 2 (*Turicibacter*, *Romboutsia*, *Enterorhabdus*, and *Bifidobacterium)*, *Turicibacter* exhibited a significant negative correlation with both variables (*p* < 0.05), supporting a potential negative synergistic effect. The other genera in this group displayed similar trends, though without statistical significance. Group 3 (*Dubosiella*, *Acetatifactor*, A2, *Ruminococcus*, and *Intestinimonas*) suggested an antagonistic effect, with abundance negatively associated with anthocyanin content and positively with mineral content. Notably, *Acetatifactor* showed a significant positive correlation with minerals (*p* < 0.01). Finally, Group 4 (*Ligilactobacillus*) showed opposite trends for minerals and anthocyanins. Overall, these findings provide preliminary insights into how mineral and anthocyanin content may modulate bacterial abundance, with some taxa showing robust associations while others require further investigation. Due to its high concentration in cranberry juice, potassium (among other minerals) has the most significant impact on the previously observed correlations. The effect of each individual mineral was assessed for further investigation. The results for potassium were similar to those obtained for the total mineral content. Additionally, the four bacterial groups identified in the total mineral content analysis were consistent across individual minerals.

In Group 1, *Colidextribacter and Oscillibacter* increased with higher mineral and anthocyanin contents. The literature did not specify the combined effect of both components on the relative abundance of these bacteria. In particular, not many studies were found on the impact of potassium, but an in silico study showed the importance of potassium for the growth of four bacterial species [36], while another study examined the relationship between potassium intake, the sodium-to-potassium (Na/K) ratio in Chinese diets, and the gut microbiota [21]. Indeed, in this study, potassium was linked to beneficial bacteria, such as *Lachnospiraceae* [21]. Another study examining diets with varying sodium (Na^+^) and potassium (K^+^) levels suggested that these minerals influence host physiology by modulating the gut microbiome. Notably, a high-Na^+^, low-K^+^ diet was associated with a significant decrease in *Porphyromonadaceae* and an increase in *Prevotellaceae* [37]. Furthermore, supplementation with coconut water (rich in potassium) affected the gut microbiota in another study [22]. The authors reported an increase in beneficial genera, including *Blautia*, *Catenibacterium*, *Eubacterium*, *Faecalibacterium*, *Ligilactobacillus*, *Prevotella*, and *Roseburia*, following supplementation and a decrease in detrimental bacteria such as *Romboutsia*. This effect, observed in patients with mild-to-moderate ulcerative colitis, may be partially attributed to potassium intake. Although the methodologies of these studies and the present study differ, they all provide initial evidence of mineral-induced gut microbiota modulation. Beneficial bacteria such as *Ligilactobacillus*, as well as potentially detrimental ones like *Romboutsia*, which were modulated in previous studies, were also detected in the present study.

On the other hand, studies highlighted the cross-talk between intestinal cells and the microbiota [38]. Any perturbations in microbial–host homeostasis could result in a shift in the composition of the microbiota. Hence, host–microbe cross-talk is strongly influenced by epithelial ion transport, and disturbances in this equilibrium can shift microbial composition [38]. Potassium supports epithelial function primarily through Na^+^/K^+^-ATPase activity, which drives Na^+^/H^+^ exchangers essential for salt, fluid, and acid–base homeostasis, as well as nutrient absorption [39]. Potassium levels may therefore indirectly shape the gut microbiota by modifying epithelial transport processes. Potassium also directly affects bacteria by regulating membrane potential, osmotic balance, and pH, and by supporting growth and division. Previous findings [40] showed that *Campylobacter jejuni* infection reduces Na^+^/K^+^-ATPase activity by 58%, leading to Na^+^ and Cl^−^ secretion and decreased glucose absorption. This disruption of the electrochemical gradient may favor dysbiosis by altering nutrient availability and epithelial barrier function. Details on the influence of K^+^ on such exchange are beyond the scope and limit of the present article, but together, these data highlighted the importance of potassium intake in microbial studies.

More studies have focused on the impact of anthocyanins on the microbiota. For *Oscillibacter*, mice treated with anthocyanins from *Lycium ruthenicum Murray* had lower relative abundances of *Oscillibacter* than in dextran sodium sulfate-induced colitis mice [41]. But, *Oscillibacter* was also highlighted as beneficial (known to be implicated in the bile acid and fat metabolism regulation), and a blackberry anthocyanin-rich extract (BE) increased the abundance of *Oscillobacter* independently of the diet’s fat content [42]. In another study, the authors highlighted that cranberry powder repressed the HFHS-induced pathobionts of *Oscillibacter* [43]. The anthocyanin extract incorporated into the diet does not contain added minerals. The discrepancy between some previous studies and the present study, which found a positive correlation between anthocyanins and *Oscillibacter* abundance, could be explained by the mineral content, but also by the differences in anthocyanin source, dosage, and experimental conditions. However, the latest studies [44] supported the beneficial impact of *Oscillibacter* and its implication in cholesterol metabolism. Concerning *Colidextribacter*, a study showed that mice treated with anthocyanin had a lower relative abundance of *Colidextribacter* than the HFD group [45]. Also, the study suggested that a decrease in *Colidextribacter* was positively correlated with unconjugated bile acid. Again, the discrepancy between this previous study and the present study, which found a positive correlation between anthocyanins and *Colidextribacter* abundance, could be explained by the different experimental conditions.

In Group 2, *Turicibacter*, *Romboutsia*, *Enterorhabdus*, and *Bifidobacterium* decreased as mineral and anthocyanin contents increased. The results on *Bifidobacterium* gain in the −31% CJ-treated group were surprising, as the positive impact of anthocyanin supplementation on its abundance is well known [15]. Similarly, mice fed with an HFHS diet and a blueberry extract were shown to have increased *Turicibacter* abundance [46], which is not what was found in the present result, which showed a statistically significant negative correlation with both minerals and anthocyanins (*p* < 0.05), supporting a negative synergistic effect. A study on rats and humans showed an increased abundance of *Turicibacter* with Davidson’s plum or dark sweet cherry consumption (containing anthocyanins), respectively, which was associated with an improvement in the symptoms of the metabolic syndrome [47,48]. The mineral content was not specified, but since sweet cherries and plums naturally contain minerals, they would be present in the supplementation but would not vary. It is important to note that *Turicibacter* is a genus of bacteria with a complex role in the gut microbiota. Indeed, *Turicibacter* can have diverse effects on the bile acids and lipid metabolism depending on the strain [49]. Regarding *Rombutsia*, a study highlighted that cranberry powder repressed the HFHS-induced pathobionts of *Romboutsia* [43]. Another study showed that a cyanidin-3-O-glucoside treatment was associated with a decrease in its relative abundances, improving the intestinal microbial dysbiosis that occurred in chronic alcohol-exposed mice [50]. In another study, the abundance of *Romboutsia* increases under an HSHF diet compared to a control diet. However, there is no significant difference in *Romboutsia* abundance between the control diet and the control diet supplemented with anthocyanins [51]. A study has shown a decrease in *Rombtousia* abundance in clinical colitis patients with coconut water supplementation (rich in dietary potassium) [22], which is in agreement with the present findings. There is a lack of information in the literature regarding the modulation of the abundance of *Enterorhabdus* associated with anthocyanin supplementation.

In Group 3, *Dubosiella*, *Acetatifactor*, *A2*, *Ruminococcus*, *and Intestinimonas* decreased with higher anthocyanin content, while their abundances increased with higher mineral content. Previous studies were in accordance with the present negative correlation of anthocyanin and *Intestinimonas* or *Acetatifactor.* Indeed, the relative abundance of *Intestinimonas* decreased in C57BL/6 J mice fed a high-fat diet supplemented with a high dose of cranberry anthocyanin extracts [16], and the results reflected a positive correlation between LPS and *Intestinimonas*. Another study showed that *Intestinimonas* decreased in mice supplemented with red raspberry (rich in anthocyanins) compared to mice with ethanol supplementation [52]. Similarly, blackberry decreased *Acetatifactor* abundance [53], generally associated with disease, which is consistent with the present observations. Conversely, a previous study did not highlight a negative correlation between anthocyanin and *Dubosiella.* Indeed, a prebiotic effect of cranberry polyphenols on *D. newyorkensis* was highlighted and characterized as a newly identified bacterial biomarker with potential to combat metabolic diseases [43]. Finally, the present findings on the negative correlation of anthocyanin and *Ruminococcus* were not in agreement with what can be found in the literature. Indeed, an HFHS diet was associated with a decrease in the abundance of *Ruminococcus* [54]. However, when anthocyanin supplementation was introduced, there was an observed increase in the abundance of *Ruminococcus* in rats [55]. *A2* is part of the *Lachnospiraceae* family. Bacteria in this family are known to increase with anthocyanin content. A study on deacidified cranberry juice showed that higher polyphenol content led to an increase in bacteria of the *Lachnospiraceae* family [5]. Other studies were in agreement with these results for polyphenol-rich cranberry extract and a bilberry anthocyanin extract [30,56]. However, details on *A2* are not available in the literature.

And finally, in Group 4, *Ligilactobacillus* increased with higher anthocyanin content, while its abundance decreased with higher mineral content. The positive correlation between *Ligilactobacillus* and anthocyanins was already demonstrated in the literature [15].

The present study was performed using healthy mice, as compared to most previous studies, which were mostly carried out in mouse models of obesity and insulin resistance, thus having gut dysbiosis and associated intestinal inflammation. This distinction likely contributes to the differences observed between our findings and earlier studies. Additionally, the interplay between minerals and anthocyanins, whether synergistic or antagonistic, may have influenced the outcomes and provided new insights. Furthermore, most of the associations reported here reflect emerging trends. While this limits the strength of the conclusions, the findings remain valuable for this first demonstration, emphasizing the importance of considering both minerals and well-known bioactives such as anthocyanins in shaping gut microbiota, even in a healthy context.

### 3.4. Distinct Composition of −31% CJ Drives Most of the Functional Shifts Observed in the Gut Microbiome

Among the pathways altered by the different juices compared to the control (water), some were also observed when comparing the juices to each other, which allows us to understand how the concentration of anthocyanins and minerals affects the gut microbiota functions.

The pathway associated with the heme biosynthesis II (anaerobic) was more represented in the gut microbiota of the control group compared to the gut of the 26% anthocyanin (Anth)/−32% mineral (Min) CJ-treated mice. But it was also more represented in the gut of the −31% Anth/−85% Min CJ-treated mice than in the gut of the 0% Anth/0% Min CJ-treated mice. Given these results, it seems this pathway was more represented when the anthocyanin concentration was low. As the water and −31% Anth/−85% Min CJ were the most demineralized, this pathway was more represented when the concentration of minerals was low as well. Similarly, the pathway associated with thiamin salvage II was more represented in the gut microbiota of the control group compared to the gut of the 26% Anth/−32% Min and 44% Anth/−60% Min CJ-treated mice. But it was also more abundant in the gut of the −31% Anth/−85% Min CJ-treated mice than in the gut of the 44% Anth/−60% Min CJ-treated mice. Given these results, it seems this pathway was more represented when the anthocyanin and mineral concentrations were low. In the same way, the L-aspartate biosynthesis superpathway was enriched in the gut microbiota of the control group compared to the gut of the 26% Anth/−32% Min CJ-treated mice. But it was also more represented in the gut of the −31% Anth/−85% Min CJ-treated mice than in the gut of any CJ-treated mice. Given these results, it seems this pathway was more abundant when the anthocyanin and mineral concentrations were low. Finally, the nucleotide biosynthesis pathway was increased in the gut microbiota of the −31% Anth/−85% Min CJ-treated mice compared to the 26% Anth/−32% Min and the −19% Anth/−70% Min CJ-treated mice. The −31% Anth/−85% Min CJ is the most impoverished and demineralized juice of the range. Thus, the nucleotide biosynthesis pathway seemed to be more represented when the anthocyanin and mineral concentrations were low.

Conversely, the pathway associated with nucleotide degradation (adenosine, guanosine, and purine nucleobases) was enriched in the gut microbiota of the 44% Anth/−60% Min CJ-treated mice compared to the control group and also compared to the −31% Anth/−85% CJ-treated mice. It appeared that nucleotide degradation was more represented when the concentration of anthocyanins was high. Similarly, the glucarate degradation pathway was more represented in the gut of the −19% Anth/−70% Min CJ-treated mice compared to the control group (water). But it was also more abundant in the gut of the 0% Anth/0% Min, −19% Anth/−70% Min, and 26% Anth/−32% Min CJ-treated mice compared to the −31% Anth/−85% Min CJ-treated mice. Results suggested that the representation of this pathway is more important for high anthocyanin concentration. Also, the teichoic acid (poly-glycerol) biosynthesis pathway was more represented in the gut microbiota of the 44% Anth/−60% Min CJ-treated mice compared to the control group (water) and also compared to the 26% Anth/−32% CJ-treated mice. Thus, the results suggest that this pathway was more prominent when anthocyanin concentrations were high. Interestingly, a significant difference was only observed between the 44% Anth/−60% Min CJ-treated mice and the 26% Anth/−32% Min CJ-treated mice, but not with the other, more impoverished juices or the more demineralized juices. The results implied that mineral concentrations would also influence the outcome of its representation. The formaldehyde assimilation II (RuMP cycle) pathway and the NAD salvage pathway II were more abundant for the −19% Anth/−70% and 44% Anth/−60% Min CJ-treated mice compared to the control group. Thus, results suggested that these pathways were more represented with a juice treatment but were not directly correlated to a higher concentration of anthocyanins. Mineral concentration seemed to impact the representation of these pathways, as the −19% Anth/−70% CJ and 44% Anth/−60% Min CJ have close percentages of demineralization. The catechol degradation I pathway was more represented in the gut microbiota of the control group compared to the −31% Anth/−85% Min and 44%/−60% Min CJ-treated mice. But it also increased in the gut of the 0% Anth/0% Min and −19% Anth/−70% CJ-treated mice than in the gut of the −31% Anth/−85% Min CJ-treated mice. It seems that this pathway representation depends on the concentrations of both anthocyanins and minerals.

Overall, pathways related to nucleotide degradation and fermentation pathways (butanoate and acetone) were more abundant in mice treated with all the juices compared to the −31% Anth/−85% Min juice or water. While ubiquinol biosynthesis and aromatic compound degradation pathways were enriched in mice treated with all the juices compared to the −31% Anth/−85% Min juice. The fermentation pathway increases, leading to the production of short-chain fatty acids (SCFAs), which is consistent with the literature, as anthocyanin supplementation has been shown to enhance SCFA production [17,57]. Also, functions associated with the butanoate metabolism were shown to be affected by the blueberry polyphenol fraction [31]. Furthermore, in a previous study, an 80% deacidified CJ induced the activation of a pathway for producing precursor metabolites and energy, such as the fermentation of pyruvate to isobutanol [5]. In contrast, functions associated with menaquinol, amino acid, and nucleotide biosynthesis were more pronounced in mice treated with the −31% Anth/−85% Min juice. Additionally, functions associated with carbohydrate degradation and lactic acid fermentation were more prominent in mice treated with the −31% Anth/−85% Min CJ compared to all the juices. The −31% Anth/−85% Min juice, characterized by its substantial reduction in anthocyanins and minerals, induced the most extensive functional alterations in the gut microbiome, suggesting that its specific composition was a key driver of these changes. Some of these functional shifts—such as increased amino acid (e.g., L-lysine biosynthesis) and nucleotide biosynthesis (e.g., adenosine), as well as enhanced carbohydrate degradation (e.g., galactose) pathways, but also functions associated with the generation of precursor metabolites and energy (pentose phosphate pathway)—were previously reported in a study on healthy mice supplemented with deacidified CJ [5]. The current findings extend beyond prior observations by highlighting other functional alterations (such as menaquinol biosynthesis and lactic acid fermentation) and the distinct impact of the highly demineralized and anthocyanin-depleted juice on gut microbial functions. Currently, no studies have reported the effects of potassium on gut microbiome alterations. However, the influence of other micronutrients on gut microbiota functions is well-documented. For example, zinc deficiency suppresses bacterial expression of pathways related to mineral absorption (e.g., zinc), carbohydrate digestion, and fermentation, leading to decreased short-chain fatty acid (SCFA) production [58]. In female mice, manganese exposure has been associated with increased microbial markers of tryptophan and phenylalanine synthesis [58]. Additionally, high dietary copper intake in piglets has been shown to alter key metabolic pathways, including protein biosynthesis, gluconeogenesis, amino acid metabolism, galactose metabolism, and the production of protein and carbohydrate metabolites [58]. Such discoveries confirmed the potential coupled effect of anthocyanins and minerals on the gut microbiome.

A limitation of the current study was the use of a simple equation to determine correlations between anthocyanins, minerals, and microbial abundance. While this approach was useful for this initial demonstration, it may have restricted the depth of the analysis and contributed to associations that represent emerging trends. Also, the observed shifts in microbial composition and predicted functions may be influenced by alternative factors, such as baseline microbiota variation or host-specific responses. Another limitation of the current study is that we used 16S rRNA gene sequencing, which allows identification primarily at the bacterial family or genus level and provides only inferred functional predictions through bioinformatic tools such as PICRUSt. While sufficient for this initial demonstration, this approach cannot capture species or strain level differences or directly measure microbial metabolic activity. Also, the study was conducted in healthy mice, whereas most previous research focused on models of obesity or insulin resistance, which may explain differences with earlier findings. Finally, although dietary potassium and anthocyanins appear to modulate the microbiota, future studies using targeted in vitro approaches on epithelial cells and bacterial cultures would help clarify causal mechanisms. Despite these limitations, the study provides valuable exploratory insights into the combined influence of minerals and anthocyanins on the gut microbiota, even in a healthy context.

## 4. Materials and Methods

### 4.1. Cranberry Juice Composition

A pasteurized and clarified cranberry juice (CJ) with a soluble solid content of 8°B was provided by Fruit d’Or (Plessisville, Quebec, Canada). Electrodialysis with filtration membranes (EDFM) was used to enrich or deplete the juices in anthocyanin at different levels: −31, −19, 0, 26, and 44%. The electrodialysis cell used in the experiment was a microflow cell (ElectroCell AB, Karlskoga, Sweden), with the cell configuration and protocol described by [24]. All CJs were stored at −20 °C and thawed at 4 °C prior to each experiment. Anthocyanin, PACs, and mineral determination were provided by [24] and are detailed in Table 3. The percentages of enrichment and demineralization of the juice are summarized in Table 4. In the subsequent sections of the article, the percentage of anthocyanin enrichment will be written only to simplify the identification.

### 4.2. Animals and Dietary Treatments

Seventy-two female C57BL/6J mice (6 weeks old) were purchased from the Jackson Laboratory (Bar Harbor, ME, USA) and housed in groups of three per cage at an ambient temperature of 22 °C, with a 12 h light/dark cycle. The mice had ad libitum access to food and water. Houses and nesting materials were provided to increase material diversity, enhance nest-building behavior, and improve overall animal comfort.

**Figure 11:**
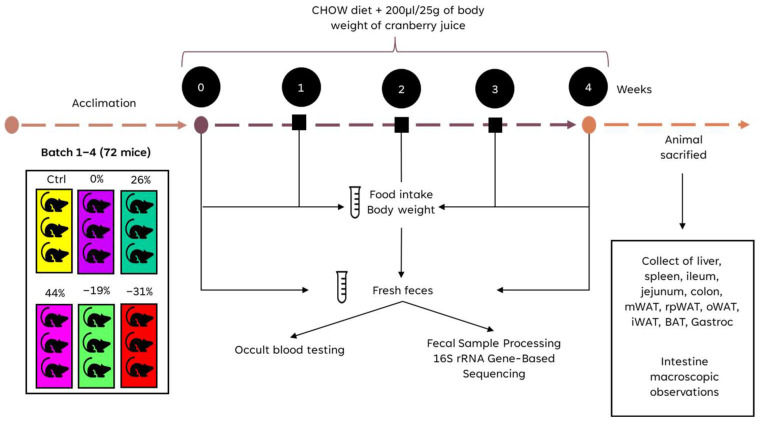
Schematic representation of experimental groups and diet treatments.

After a two-week acclimation period, the mice were randomly divided into six groups (control group with water, and 0%, −31%, −19%, 26%, and 44% CJ groups) with 12 mice per group, balanced according to their initial weight (18.1 to 18.2 g per group) (Figure 11). A priori calculations indicated that detecting a 20% difference in the variables under study with a standard deviation of 15% (α = 0.05 and a statistical power of 0.8) would require between 6 and 16 mice per group. Based on previous experience, a sample size of 12 mice per group was considered sufficient to reliably detect treatment effects. Cages were arranged vertically on the rack, with one cage from each experimental group assigned to a different row to account for potential variation in light exposure.

All mice were fed a standard CHOW diet and received a daily oral dose of 200 μL/25 g of body weight, either water for the control group or one of the five CJ concentrations, for four weeks. The order of treatment administration was varied each day. The volume of water or CJ was adjusted biweekly based on each mouse’s weight to ensure the maximum dose was administered. Data on mouse weight and food consumption per cage were recorded twice weekly. Fresh feces were collected in two sterile tubes at three time points: before the experiment (T0), after two weeks of gavage (T2), and before sacrifice (T4). One fecal sample was used for Hemoccult testing to detect occult blood (OB), and the other was reserved for gut microbiota analysis. After the four-week study, the mice were anesthetized using 2–3% isoflurane in chambers and randomly sacrificed via cardiac puncture. Organs, including the liver, spleen, ileum, and colon, were collected for further analysis. The present study followed the *Guide for the care and use of laboratory animals*, and the experimental protocol was approved by the Laval University Animal Ethics Committee (protocol code 2023-1378).

### 4.3. Analyses Determining Intestinal Inflammation

#### 4.3.1. Occult Blood Testing

The presence of occult blood (OB) in fresh fecal samples was detected using the Hemoccult SENSA kit (Beckman Coulter, Brea, CA, USA), following the manufacturer’s instructions.

#### 4.3.2. Intestinal Macroscopic Observations

Intestines were removed from the mice and gently rinsed with cold 1× Phosphate-Buffered Saline (PBS) before the isolation of each intestinal part (duodenum, ileum, jejunum, and colon). Based on the existing literature [5], a severity score for intestinal inflammation was assessed through macroscopic examination with a magnifying glass. A severity score, ranging from 0 to 3, was attributed for three criteria: inflammation (reddish areas), vascularization, and thickening.

### 4.4. Analyses of the Gut Microbiota

#### 4.4.1. Fecal Sample Processing and 16S rRNA Gene-Based Sequencing

Fresh feces collected at T4 were used for the analysis. The microbiota composition of gut contents was determined by 16S rRNA amplicon sequencing. Fecal DNA was extracted from fresh feces with the NucleoSpin^®^96 Soil kit (Macherey-Nagel, Duren, Germany) and then sent to the sequencing platform at Génome Québec for PCR amplification of the V3-V4 region using the primers 341F and 805R, followed by sequencing on an Illumina NextSeq sequencing platform (Illumina, San Diego, CA, USA). DNA quality and yield were sufficient to ensure successful amplification and sequencing of all samples.

#### 4.4.2. Gut Microbiota Analyses and Functional Prediction of the Gut Bacterial Communities

Forward and reverse primers were removed from raw paired-end reads using Cutadapt (v4.1). Sequences were processed using the DADA2 package (v1.30) [59] in the R environment (http://www.R-project.org; accessed on 18 July 2024). Reads with an expected error threshold > 2 and >4 for the forward and reverse reads, respectively, with ambiguous bases, and with quality scores less than or equal to 2 were discarded. Dereplication and denoising of filtered sequences were carried out using DADA2 default parameters. Associations with bacterial taxa were obtained using the RDP classifier algorithm (v2.2) [60] trained against the Silva database 138.1 [61]. After sample rarefaction to an even sampling depth of 38 258 sequences to normalize for sequencing depth, bacterial α-diversity was calculated using the Shannon and Simpson’s reciprocal indexes [62]. Principal component analysis (PCA) was performed on the Aitchison distance matrix in order to measure β-diversity [63]. A graphical visualization of α- and β-diversity results was achieved using the ‘phyloseq’ R package (version 1.46.0). Identification of differentially abundant bacteria or pathways between two distinct biological conditions was measured with DESeq2 [25]. The functional composition of bacterial communities was predicted using the Phylogenetic Investigation of Communities by Reconstruction of Unobserved States 2 (PICRUSt2) pipeline [64]. The predicted functions were collapsed into MetaCyc pathways [65].

### 4.5. Statistical Analysis

A one-way ANOVA was used to assess differences between groups, and Tukey’s post hoc test was applied when the ANOVA indicated statistically significant results (*p* < 0.05). Sigma software (v12.0) was utilized for this statistical analysis, and significant differences were determined at a probability level of *p* < 0.05.

Differential abundance analysis was performed using DESeq2, which normalizes raw read counts to account for sequencing depth and performs statistical testing using a negative binomial distribution to model observed absolute abundances. After model fitting, the Wald test is used to evaluate the taxonomic differences between groups.

To assess the correlation between bacterial abundance and the percentage of mineral and anthocyanin enrichment, dynamic curve fitting was performed using SigmaPlot (v12.0). For the curve fitting, a plane equation (Z = z_0_ + ax + by) was selected to model the relationship between bacterial abundance and the enrichment percentages of minerals and anthocyanins. The dynamic curve fit method was applied with automatic computation to determine the best fit for the data. The software automatically computed the optimal parameters for the plane equation to best describe the observed data.

## 5. Conclusions

The present study is the first to assess the modulation of gut microbiota composition in healthy mice by cranberry juice, specifically focusing on the combined actions of dietary anthocyanins and minerals. While the alpha- and beta-diversity of the gut microbiota remained unchanged between groups at the end of the study, differential abundance analysis revealed variations based on anthocyanin and mineral enrichments. The study clustered genera according to the potential combined positive or negative impacts of minerals and anthocyanins, revealing possible synergistic and antagonistic effects. Predicted functions related to nucleotide degradation and fermentation (butanoate and acetate) were more prevalent in mice treated with the anthocyanin-rich juice. Interestingly, the distinct composition of −31% anthocyanin/−85% mineral CJ drives the most profound functional alterations in the gut microbiome. There was no sign of inflammation induced by the organic acid in impoverished juices. Thus, the modification of cranberry juice by EDFM facilitates its incorporation into diets, offering a practical alternative to supplementation. Future studies on altered gut microbiota of animal models of intestinal and metabolic diseases or humans could provide more information on the effects of various concentrations of minerals (e.g., potassium) and anthocyanins on key gut and metabolic endpoints.

## Figures and Tables

**Table 1:** Effect of cranberry juice (CJ) anthocyanin content on the inflammatory state of the duodenum, jejunum, ileum, and the colon. Inflammation, thickness, and vascularization were scored from 0 to 3 based on macroscopic observation. Results are presented as means ± SD for each group at each column (*n* = 12). Statistical analysis was performed using one-way ANOVA followed by Tukey’s post hoc test (*p* < 0.05). A different letter in this column indicates a statistically significant difference between the groups at a probability level of *p* < 0.05.

	Duodenum			Jejunum			Ileum			Colon		
	Inflammation	Thickness	Vascularization	Inflammation	Thickness	Vascularization	Inflammation	Thickness	Vascularization	Inflammation	Thickness	Vascularization
**Control**	0 ± 0 ^a^	0 ± 0 ^a^	0.250 ± 0.452 ^a^	0 ± 0 ^a^	0 ± 0 ^a^	0.083 ± 0.289 ^a^	0 ^a^ ± 0 ^a^	0 ± 0 ^a^	0 ± 0 ^a^	0 ± 0 ^a^	0 ± 0 ^a^	0 ± 0 ^a^
**0% CJ**	0 ± 0 ^a^	0.250 ± 0.452 ^a^	0.583 ± 0.515 ^a^	0 ± 0 ^a^	0.083 ± 0.289 ^a^	0.167 ± 0.389 ^a^	0 ^a^ ± 0 ^a^	0.083 ± 0.289 ^a^	0.083 ± 0.289 ^a^	0 ± 0 ^a^	0 ± 0 ^a^	0 ± 0 ^a^
**24% CJ**	0 ± 0 ^a^	0.333 ± 0.492 ^a^	0.833 ± 0.389 ^a^	0 ± 0 ^a^	0.167 ± 0.389 ^a^	0.083 ± 0.389 ^a^	0 ^a^ ± 0 ^a^	0 ± 0 ^a^	0.083 ± 0.289 ^a^	0 ± 0 ^a^	0 ± 0 ^a^	0.083 ± 0.289 ^a^
**44% CJ**	0.083 ± 0.289 ^a^	0.250 ± 0.452 ^a^	0.750 ± 0.452 ^a^	0 ± 0 ^a^	0.083 ± 0.289 ^a^	0.167 ± 0.452 ^a^	0 ^a^ ± 0 ^a^	0 ± 0 ^a^	0.083 ± 0.289 ^a^	0 ± 0 ^a^	0 ± 0 ^a^	0 ± 0 ^a^
**−19% CJ**	0 ± 0 ^a^	0.417 ± 0.515 ^a^	0.583 ± 0.515 ^a^	0 ± 0 ^a^	0.083 ± 0.289 ^a^	0.167 ± 0.389 ^a^	0 ± 0 ^a^	0 ± 0 ^a^	0 ± 0 ^a^	0 ± 0 ^a^	0 ± 0 ^a^	0 ± 0 ^a^
**−31% CJ**	0 ± 0 ^a^	0.333 ± 0.667 ^a^	0.492 ± 0.492 ^a^	0 ± 0 ^a^	0.083 ± 0.289 ^a^	0.167 ± 0.389 ^a^	0 ± 0 ^a^	0 ± 0 ^a^	0.333 ± 0.492 ^a^	0 ± 0 ^a^	0 ± 0 ^a^	0 ± 0 ^a^

**Table 2:** Regression coefficients of the plane equation (Z = z_0_ + ax + by). Coefficient a represents the effect of x (anthocyanin), while coefficient b corresponds to the effect of y (minerals or potassium).

		*All minerals*								*K*			
		*R^2^*	*z_o_*	*a*	*p*-Value	*b*	*p*-Value	*R^2^*	*z_o_*	*a*	*p*-Value	*b*	*p*-Value
**Group 1**	*Colidextribacter*	0.74	3.893	7.12 × 10^−4^	0.748	3.64 × 10^−3^	0.177	0.73	3.892	1.171 × 10^−3^	0.599	3.318 × 10^−3^	0.183
	*Oscillibacter*	0.475	4.061	6.76 × 10^−4^	0.865	3.80 × 10^−3^	0.360	0.47	4.06	1.159 × 10^−3^	0.767	3.447 × 10^−3^	0.368
**Group 2**	*Turicibacter*	0.96	3.782	−3.29 × 10^−3^	0.042	−2.36 × 10^−3^	0.0467	0.96	3.782	−3.588 × 10^−3^	0.036	−2.157 × 10^−3^	0.071
	*Romboutsia*	0.80	2.902	−3.53 × 10^−3^	0.277	−3.73 × 10^−3^	0.231	0.79	2.902	−4.00 × 10^−3^	0.228	−3.395 × 10^−3^	0.238
	*Enterorhabdus*	0.70	2.536	−1.02 × 10^−3^	0.513	−1.90 × 10^−3^	0.250	0.69	2.537	−1.258 × 10^−3^	0.423	−1.730 × 10^−3^	0.257
	*Bifidobacterium*	0.56	3.674	−2.96 × 10^−3^	0.511	−3.44 × 10^−3^	0.423	0.55	3.675	−3.393 × 10^−3^	0.448	−3.131 × 10^−3^	0.427
**Group 3**	*Dubosiella*	0.603	3.077	−1.13 × 10^−2^	0.224	3.81 × 10^−3^	0.590	0.60	3.075	−1.086 × 10^−2^	0.227	3.455 × 10^−3^	0.595
	*Acetatifactor*	0.88	3.244	−7.41 × 10^−4^	0.644	4.63 × 10^−3^	0.067	0.87	3.244	−1.615 × 10^−4^	0.916	4.234 × 10^−3^	0.071
	*A2 (Lachnospiraceae)*	0.60	3.492	−6.84 × 10^−3^	0.246	4.48 × 10^−3^	0.368	0.60	3.491	−6.272 × 10^−3^	0.266	4.074 × 10^−3^	0374
	*Ruminococcus*	0.82	3.45	−2.24 × 10^−3^	0.281	4.26 × 10^−3^	0.094	0.81	3.450	−1.709 × 10^−3^	0.377	3.889 × 10^−3^	0.099
	*Intestinimonas*	0.475	3.797	−1.58 × 10^−3^	0.671	3.97 × 10^−3^	0.311	0.46	3.796	−1.075 × 10^−3^	0.764	3.602 × 10^−3^	0.319
**Group 4**	*Ligilactobacillus*	0.61	2.765	3.46 × 10^−3^	0.552	−7.86 × 10^−3^	0.222	0.59	2.767	2.464 × 10^−3^	0.658	−7.145 × 10^−3^	0.230

**Table 3:** Juice physicochemical properties. Values followed with different letters (a, b, c, d, and e) for the same line are significantly different (ANOVA and Tukey’s test) at *p* < 0.05. * The initial juice composition represents the average of each repetition of the juices within the system prior to treatment, ** the statistical test was applied on transformed data, *** test with rank transformation.

Properties		Juice Identification				
		Initial Juice *	EDFM 3 h Enriched Juice	EDFM 6 h Enriched Juice	EDFM 3 h Raw Juice	EDFM 6 h Raw Juice
Anthocyanin (mg/L)	Cyanidin-3-galactoside	40.99 ± 0.25 ^a^**	51.88 ± 1.11 ^b^**	59.34 ± 2.29 ^c^**	33.2 ± 0.66 ^d^**	28.3 ± 0.66 ^e^**
	Cyanidin-3-glucoside	1.4 ± 0.11 ^a^	1.69 ± 0.06 ^b^	1.96 ± 0.1 ^c^	1.12 ± 0.02 ^d^	0.99 ± 0.03 ^d^
	Cyanidin-3-arabinoside	40.20 ± 0.43 ^a^	50.6 ± 1.17 ^b^	57.87 ± 2.48 ^c^	32.45 ± 0.67 ^d^	27.69 ± 0.67 ^e^
	Peonidin-3-galactoside	60.29 ± 0.49 ^a^**	75.64 ± 1.6 ^b^**	86.37 ± 3.32 ^c^**	49.08 ± 0.93 ^d^**	42.12 ± 1.04 ^e^**
	Peonidin-3-glucoside	7.26 ± 0.26 ^a^	9.07 ± 0.27 ^b^	10.57 ± 0.27 ^c^	6.03 ± 0.13 ^d^	5.33 ± 0.29 ^e^
	Peonidin-3-arabinoside	30.95 ± 0.20 ^a^**	38.75 ± 0.89 ^b^**	44.37 ± 1.90 ^c^**	25.18 ± 0.50 ^d^**	21.61 ± 0.49 ^e^**
Proanthocyanidins (mg/L)	Monomers	29.30 ± 2.83 ^a^**	31.54 ± 1.14 ^a^**	33.03 ± 2.07 ^a^**	28.08 ± 3.02 ^a^**	29.43 ± 5.65 ^a^**
	2–3 mers	88.42 ± 1.08 ^a^	90.45 ± 3.0 ^a^	88.93 ± 0.98 ^a^	88.89 ± 3.16 ^a^	89.14 ± 2.52 ^a^
	4–5 mers	15.49 ± 0.32 ^a^	15.61 ± 0.89 ^a^	15.04 ± 0.32 ^a^	16.08 ± 1.09 ^a^	15.47 ± 0.52 ^a^
	6–7 mers	4.30 ± 0.23 ^a^	4.42 ± 0.67 ^a^	3.75 ± 0.6 ^a^	4.56 ± 0.59 ^a^	3.91 ± 0.35 ^a^
	>7 mers	0.00 ± 0.00 ^a^	0.00 ± 0.00 ^a^	0.00 ± 0.00 ^a^	0.00 ± 0.00 ^a^	0.00 ± 0.00 ^a^
Minerals (mg/L)	Ca	52.72 ± 0.21 ^a^	60.56 ± 2.78 ^b^	57.16 ± 0.45 ^b^	20.77 ± 1.20 ^c^	11.25 ± 0.99 ^d^
	Cu	0.12 ± 0.00 ^abc^***	0.16 ± 0.01 ^a^***	0.19 ± 0.01 ^b^***	0.10 ± 0.00 ^a^***	0.08 ± 0.01 ^c^***
	K	681.82 ± 2.98 ^a^	414.61 ± 20.67 ^b^	195.80 ± 20.76 ^c^	174.61 ±12.92 ^c^	78.87 ± 6.22 ^d^
	Mg	36.15 ± 0.07 ^a^	47.18 ± 3.18 ^b^	50.92 ± 2.21 ^b^	14.56 ± 0.92 ^c^	7.66 ± 0.73 ^d^
	Na	11.84 ± 0.15 ^a^	9.55 ± 0.43 ^b^	6.82 ± 0.33 ^c^	3.92 ± 0.3 ^d^	1.98 ± 0.15 ^e^
	P	35.89 ± 0.22 ^a^**	23.52 ± 0.29 ^b^**	18.58 ± 1.14 ^c^**	28.75 ± 0.55 ^d^**	24.25 ± 2.23 ^b^**

**Table 4:** Group identification, % enrichment in anthocyanins, and % demineralization of the juices.

		Group Identification				
	A	B	C	D	E	F
**% Anthocyanin enrichment**	Water (control group)	0 ± 0	26 ± 4	44 ± 5	−19 ± 1	−31 ± 3
**% Global demineralization**		0 ± 0	−32 ± 3	−60 ± 3	−70 ± 2	−85 ± 1

## Data Availability

Data is contained within the article.

## Supplementary Materials

The following supporting information can be downloaded at https://www.mdpi.com/article/10.3390/molecules30193986/s1: Figure S1: Administration of cranberry juices (CJs) with different anthocyanin concentrations is associated with changes in the gut microbial functional pathways of mice in comparison with the most anthocyanin-impoverished CJ. Figure S2. Administration of cranberry juices (CJs) with different anthocyanin concentrations is associated with changes in the gut microbial pathways in comparison with the 26% CJ.

## Abbreviations

The following abbreviations are used in this manuscript:

CJ	Cranberry juice
EDFM	Electrodialysis with filtration membrane
HFHS	High fat, high sugar
OB	Occult blood
PAC	Proanthocyanidins
