# Supplementary material for: Coupled Impact of Anthocyanin and Mineral Concentrations in Cranberry Juice on Gut Microbiota and Function Modulation: A First Demonstration
Source: Molecules. 2025 Oct 4;30(19):3986. doi: 10.3390/molecules30193986 (PMC12526315; doi:10.3390/molecules30193986)
Supplement: Supplementary file 1 [file molecules-30-03986-s001.zip › molecules-3754569-supplementary.pdf]

Supplementary material

2.4.2 Effect of anthocyanin concentration in the juice on the functional predictions of the gut microbiota

The gut microbiota of the -31% CJ-treated mice, nucleotides, amino acid, menaquinol biosynthesis (e.g., guanosine ribonucleotides de novo biosynthesis, superpathway of L-lysine, L-threonine and L-methionine biosynthesis I) pathways were more abundant compared to the 26% CJ-treated mice (Figure S1a). In parallel, aromatic compound, amino acid degradation (e.g., toluene degradation), organic acid, amino acid fermentation (pyruvate fermentation) and short chain fatty acid (SCFA) biosynthesis (acetate and butanotate) pathways were more represented in the gut microbiota of the 26% CJ-treated mice compared to the -31% CJ-treated mice (Figure S1a). Comparably, sugar degradation (e.g., glucose and glucose-1-phosphate degradation), nucleotides and amino acid biosynthesis (e.g., guanosine deoxyribonucleotides de novo biosynthesis II) and generation of precursor metabolites and energy (pentose phosphate pathways) pathways were more abundant in the gut microbiota of the -31% CJ-treated mice compared to the -19% CJ-treated mice (Figure S1b). In contrast, aromatic compound (e.g., catechol degradation I), sugar and fatty acid degradation (e.g., fatty acid β-oxidation I) and fermentation (e.g., pyruvate fermentation to butanoate) pathways were enriched in the gut microbiota of the -19% CJ-treated mice compared to the -31% CJ-treated mice, (Figure S1b).

-31% CJ

(most impoverished juice)

vs

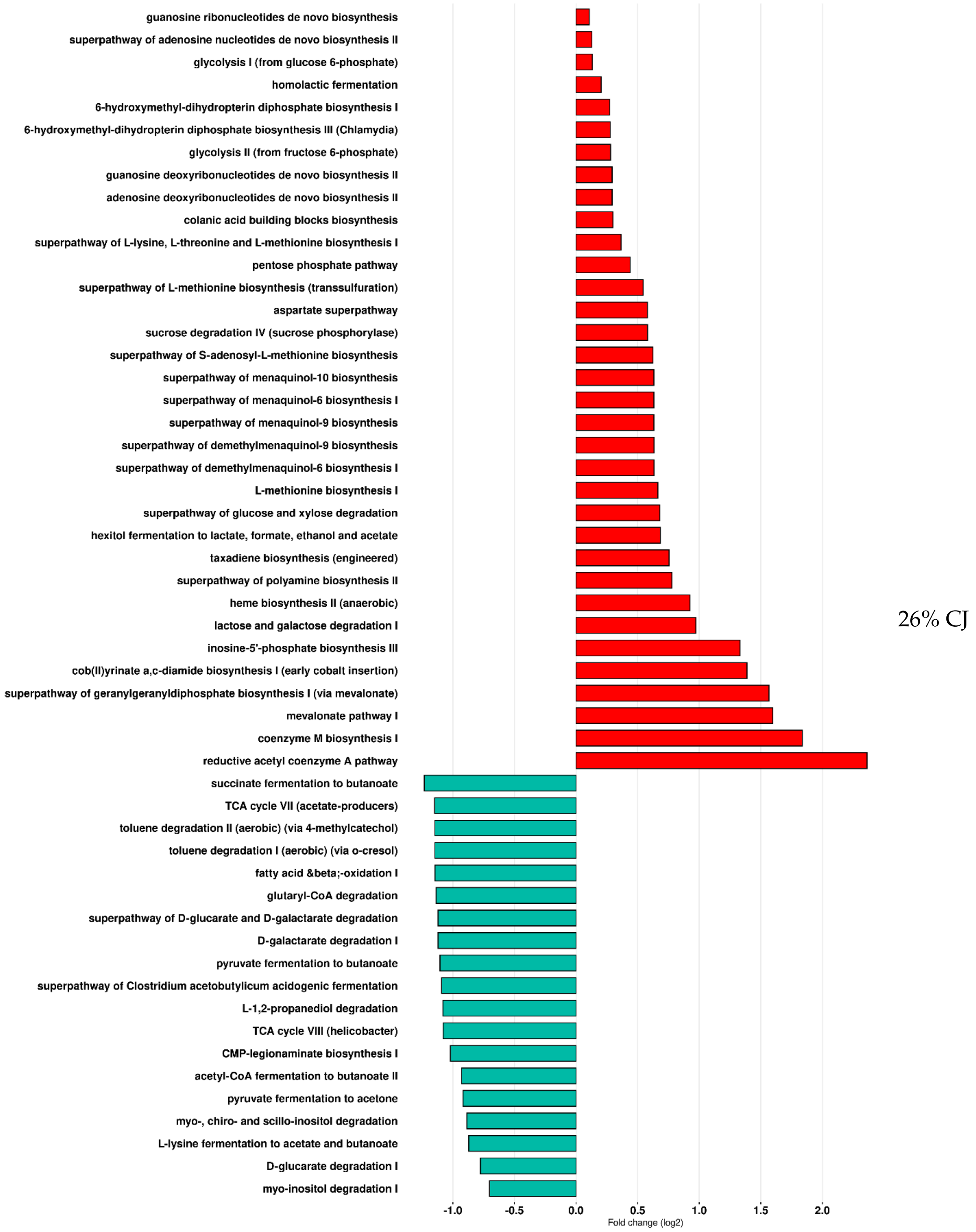
guanosine ribonucleotides de novo biosynthesis
superpathway of adenosine nucleotides de novo biosynthesis II
glycolysis I (from glucose 6-phosphate)
homolactic fermentation
6-hydroxymethyl-dihydropterin diphosphate biosynthesis I
6-hydroxymethyl-dihydropterin diphosphate biosynthesis III (Chlamydia)
glycolysis II (from fructose 6-phosphate)
guanosine deoxyribonucleotides de novo biosynthesis II
adenosine deoxyribonucleotides de novo biosynthesis II
colanic acid building blocks biosynthesis
superpathway of L-lysine, L-threonine and L-methionine biosynthesis I
pentose phosphate pathway
superpathway of L-methionine biosynthesis (transsulfuration)
aspartate superpathway
sucrose degradation IV (sucrose phosphorylase)
superpathway of S-adenosyl-L-methionine biosynthesis
superpathway of menaquinol-10 biosynthesis
superpathway of menaquinol-6 biosynthesis I
superpathway of menaquinol-9 biosynthesis
superpathway of demethylmenaquinol-9 biosynthesis
superpathway of demethylmenaquinol-6 biosynthesis I
L-methionine biosynthesis I
superpathway of glucose and xylose degradation
hexitol fermentation to lactate, formate, ethanol and acetate
taxadiene biosynthesis (engineered)
superpathway of polyamine biosynthesis II
heme biosynthesis II (anaerobic)
lactose and galactose degradation I
inosine-5'-phosphate biosynthesis III
cob(II)yrinate a,c-diamide biosynthesis I (early cobalt insertion)
superpathway of geranylgeranyldiphosphate biosynthesis I (via mevalonate)
mevalonate pathway I
coenzyme M biosynthesis I
reductive acetyl coenzyme A pathway
succinate fermentation to butanoate
TCA cycle VII (acetate-producers)
toluene degradation II (aerobic) (via 4-methylcatechol)
toluene degradation I (aerobic) (via o-cresol)
fatty acid β-oxidation I
glutaryl-CoA degradation
superpathway of D-glucarate and D-galactarate degradation
D-galactarate degradation I
pyruvate fermentation to butanoate
superpathway of Clostridium acetobutylicum acidogenic fermentation
L-1,2-propanediol degradation
TCA cycle VIII (helicobacter)
CMP-legionaminate biosynthesis I
acetyl-CoA fermentation to butanoate II
pyruvate fermentation to acetone
myo-, chiro- and scillo-inositol degradation
L-lysine fermentation to acetate and butanoate
D-glucarate degradation I
myo-inositol degradation I
-1.0
-0.5
0.0
0.5
1.0
1.5
2.0
Fold change (log2)
26% CJ

(a)

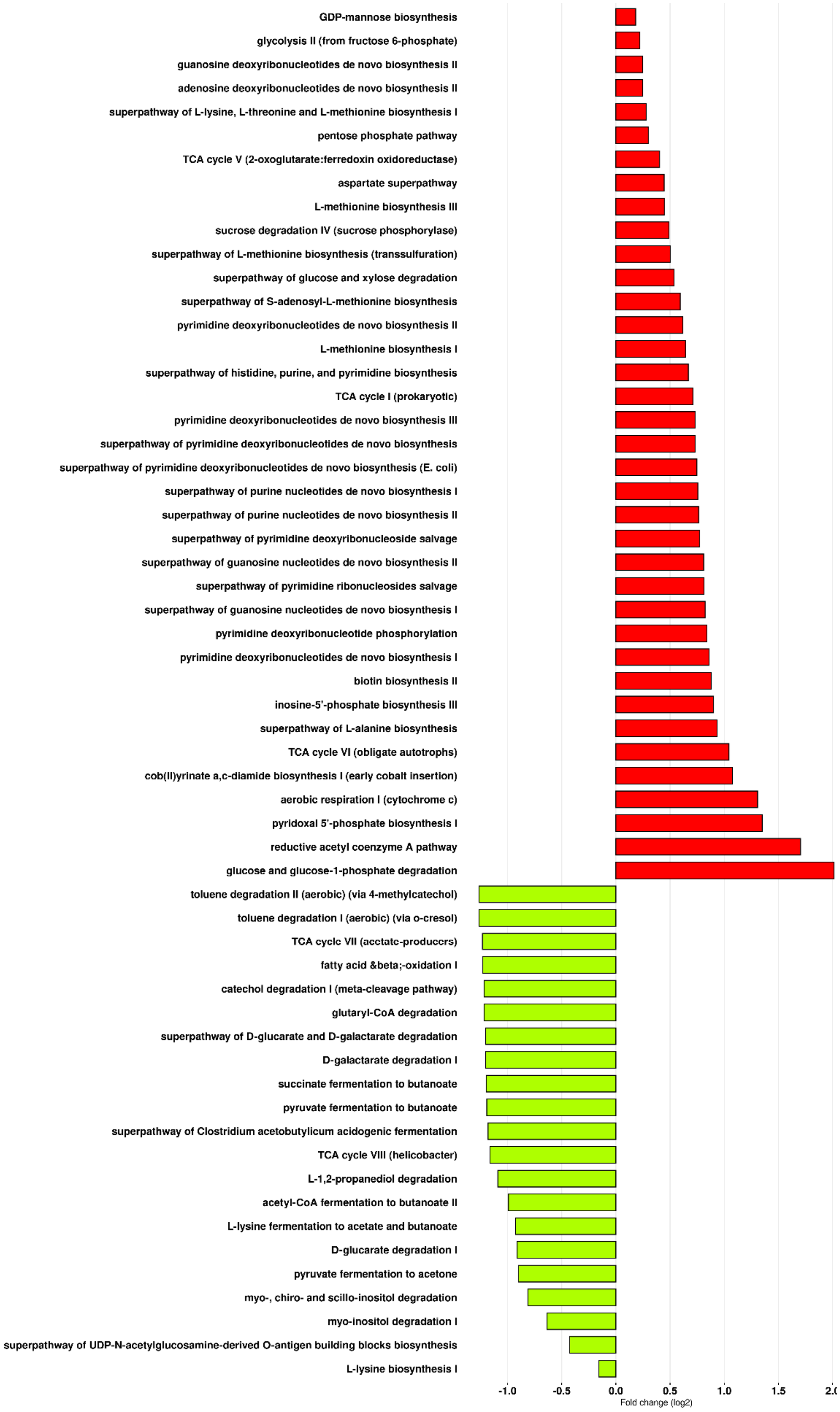

-19% CJ

(b)

Figure S1. Administration of cranberry juices (CJs) with different anthocyanin concentrations is associated with changes in the gut microbial functional pathways of mice in comparison with the most anthocyanin-

impoverished CJ. The differential abundance analysis was calculated in order to explore microbial pathways that more strongly discriminated between the gut microbiota of mice treated with (a) -31% CJ (red) and 26% CJ (blue), (b) -31% CJ (red) and -19% CJ (green).

Function of sugar degradation pathway was more represented in the 26% CJ-treated mice compared to the -19% group (Figure S2b). Pathways associated to alcohol and peptidoglycan biosynthesis were more abundant in the 44% CJ-treated mice compared to the 26% group (Figure S2a).

26% CJ

vs

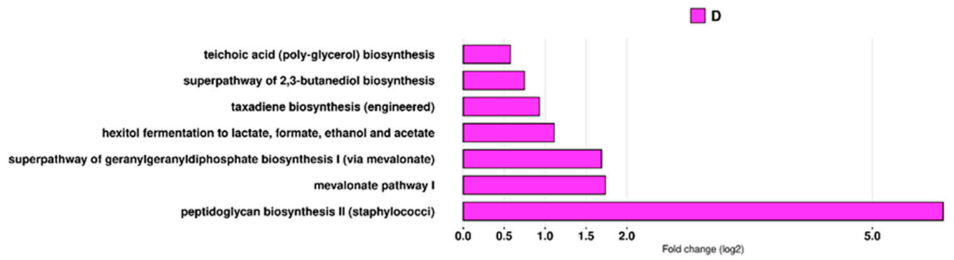

44%

(a)

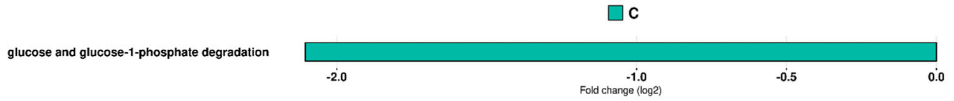

-19%

(b)

Figure S2. Administration of cranberry juices (CJs) with different anthocyanin concentrations is associated with changes in the gut microbial pathways in comparison with the 26% CJ. The differential abundance analysis was calculated in order to explore the microbial pathways that more strongly discriminated between the gut microbiota of mice treated with (a) 26% CJ (blue) and 44% CJ (pink), (b) 26% CJ (blue) and -19% CJ (green).
